# Simultaneous recordings of action potentials and calcium transients from human induced pluripotent stem cell derived cardiomyocytes

**DOI:** 10.1242/bio.035030

**Published:** 2018-07-03

**Authors:** Chandra Prajapati, Risto-Pekka Pölönen, Katriina Aalto-Setälä

**Affiliations:** 1 BioMediTech, University of Tampere, 33520 Tampere, Finland; 2 Faculty of Medicine and Life Science, University of Tampere, 33520 Tampere, Finland; 3 Heart Hospital, Tampere University Hospital, 33520 Tampere, Finland

**Keywords:** hiPSC-CMs, Membrane potential, Calcium transient, Delayed after depolarization, Early after depolarization

## Abstract

Human induced pluripotent stem cell derived cardiomyocytes (hiPSC-CMs) offer a unique *in vitro* platform to study cardiac diseases, as they recapitulate many disease phenotypes. The membrane potential (V_m_) and intracellular calcium (Ca^2+^) transient (CaT) are usually investigated separately, because incorporating different techniques to acquire both aspects concurrently is challenging. In this study, we recorded V_m_ and CaT simultaneously to understand the interrelation between these parameters in hiPSC-CMs. For this, we used a conventional patch clamp technique to record V_m_, and synchronized this with a Ca^2+^ imaging system to acquire CaT from same hiPSC-CMs. Our results revealed that the CaT at 90% decay (CaT90) was longer than action potential (AP) duration at 90% repolarization (APD90). In addition, there was also a strong positive correlation between the different parameters of CaT and AP. The majority of delayed after depolarizations (DADs) observed in the V_m_ recording were also characterized by elevations in the intracellular Ca^2+^ level, but in some cases no abnormalities were observed in CaT. However, simultaneous fluctuations in CaT were always observed during early after depolarizations (EADs) in V_m_. In summary, simultaneous recording of V_m_ and CaT broadens the understanding of the interrelation between V_m_ and CaT and could be used to elucidate the mechanisms underlying arrhythmia in cardiac disease condition.

## Abstract (precis)

**Summary:** We performed simultaneous recordings of action potentials and calcium transients from the same cardiomyocytes to improve understanding of the mechanisms underlying arrhythmias and the correlation between these two parameters.

## INTRODUCTION

The discovery of human induced pluripotent stem cells (hiPSCs) (Takahashi et al., 2007) from somatic cells and their ability to differentiate into cardiomyocytes (CMs) (hiPSC-CMs) provides a robust platform to study genetic cardiac diseases (Kujala et al., 2012; Lahti et al., 2012; Kiviaho et al., 2015; Penttinen et al., 2015; Ojala et al., 2016; Kuusela et al., 2017; Prajapati et al., 2018; Itzhaki et al., 2012; Ma et al., 2015; Spencer et al., 2014) and for drug screening (Liang et al., 2013). Since hiPSC-CMs have the same genetic information as the donor, they provide a patient-specific *in vitro* modelling set-up. At the molecular level, hiPSC-CMs express major cardiac ion channels (Lee et al., 2016), Ca^2+^ cycling components (Itzhaki et al., 2011) and adrenergic receptors (Földes et al., 2014). Thus, these hiPSC-CMs closely mimic cardiac functionality and have already recapitulated many genetic cardiac diseases such as LQT1 (Kiviaho et al., 2015; Kuusela et al., 2017; Ma et al., 2015), LQT2 (Lahti et al., 2012; Spencer et al., 2014), CPVT (Kujala et al., 2012; Itzhaki et al., 2012; Penttinen et al., 2015; Pölönen et al., 2018; Ahola et al., 2017) and HCM (Ojala et al., 2016; Prajapati et al., 2018; Földes et al., 2014; Lan et al., 2013; Han et al., 2014). In CMs, the contraction is driven by action potential (AP) induced release of intracellular Ca^2+^. Inward current through sodium channels (I_Na_) starts the rapid depolarization of the CM cell membrane, which triggers inward current through L-type calcium channels (I_Ca,L_). This leads to Ca^2+^ induced Ca^2+^ release (CICR) from the sarcoplasmic reticulum (SR) via ryanodine receptors. Ca^2+^ binds to sarcomeres, the contractile units of myofibrils, and allows the movement of myofilaments and contractile motion. The cell membrane is then repolarized by several repolarizing potassium currents and Ca^2+^ is reloaded into SR via SR Ca^2+^ ATPase. (Kane et al., 2015)

The presence of ion channels plays a major role in shaping AP and Ca^2+^ dynamics (Bartos et al., 2015). The membrane potential (V_m_) and intracellular Ca^2+^ concentration [Ca^2+^]_i_ are the most crucial elements in the normal physiology and also in arrhythmias in cardiology. Ca^2+^ ion plays a major role in activation and modulation of contraction in CMs. The measurement of [Ca^2+^]_i_ using a Ca^2+^ indicator during contraction and relaxation of CMs reflects the SR Ca^2+^ release and uptake. Furthermore, [Ca^2+^]_i_ influences the shape and duration of AP via Ca^2+^-sensitive ionic channels such as I_Ca_, Na^+^-Ca^2^+ exchanger (NCX) and Ca^2+^-activated nonselective cation channels, thereby modifying the electrophysiological properties, for example, the refractoriness and membrane depolarization rate (Wu et al., 2005; Bers, 2002). Conversely, V_m_ can influence the Ca^2+^ spark and waves (Sato et al. 2014). Thus, the V_m_ and Ca^2+^ cycle are highly interdependent and bidirectionally coupled (Omichi et al., 2004). Only few studies have been focused on simultaneous recording of V_m_ and calcium transient (CaT) from the same hiPSC-CMs (Lee et al., 2012; Spencer et al., 2014). Thus, this hinders the understanding of inter-relations between V_m_ and CaT from the same hiPSC-CMs. To improve the understanding of the complex dynamics and mechanisms underlying arrhythmias, it is ideal to analyse V_m_ and CaT simultaneously from the same cell. In this study, we performed simultaneous V_m_ and CaT recordings from the same hiPSC-CMs to investigate in more detail the correlation between these parameters.

## RESULTS

### Immunocytochemistry

**Fig. 1.**
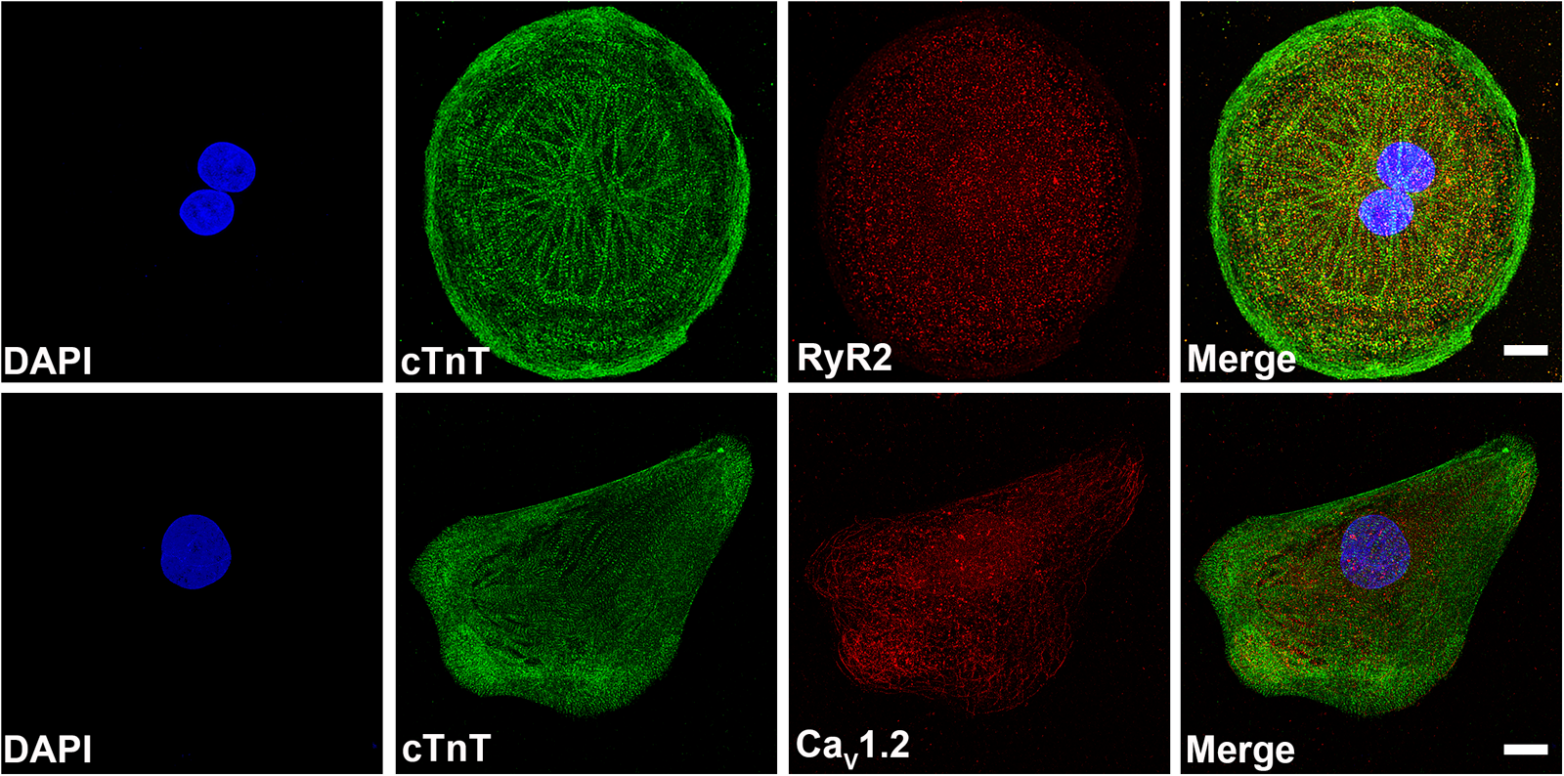
**Representative immunofluorescence images of hiPSC-CMs showing cardiac proteins.** (Upper) 50 slices were combined from confocal immunofluorescence images of nucleus (DAPI, blue), cardiac troponin T (cTnT, green) and cardiac ryanodine receptors (RyR2, red). Final panel shows the former three merged. (Lower) 40 slices were combined from confocal immunoflurescence images of nucleus (DAPI, blue), cTnT (green), L-type calcium channels (Ca_V_1.2, red). Final panel shows the former three merged. Scale bars: 10 µM.

Immunostaining experiments were performed to confirm the presence of different cardiac proteins in hiPSC-CMs. Dissociated hiPSC-CMs were imaged with a confocal microscope with z-stack. Immunostainings of hiPSC-CMs showed the positive staining of cardiac troponin T (cTnT) (green), CaV1.2 (red) and cardiac ryanodine receptor (RyR2) (red) (Fig. 1). These immunostaining images show the homogenous distribution of I_Ca,L_ and RyR2 in hiPSC-CMs.

### Voltage-gated ionic current hiPSC-CMs

**Fig. 2.**
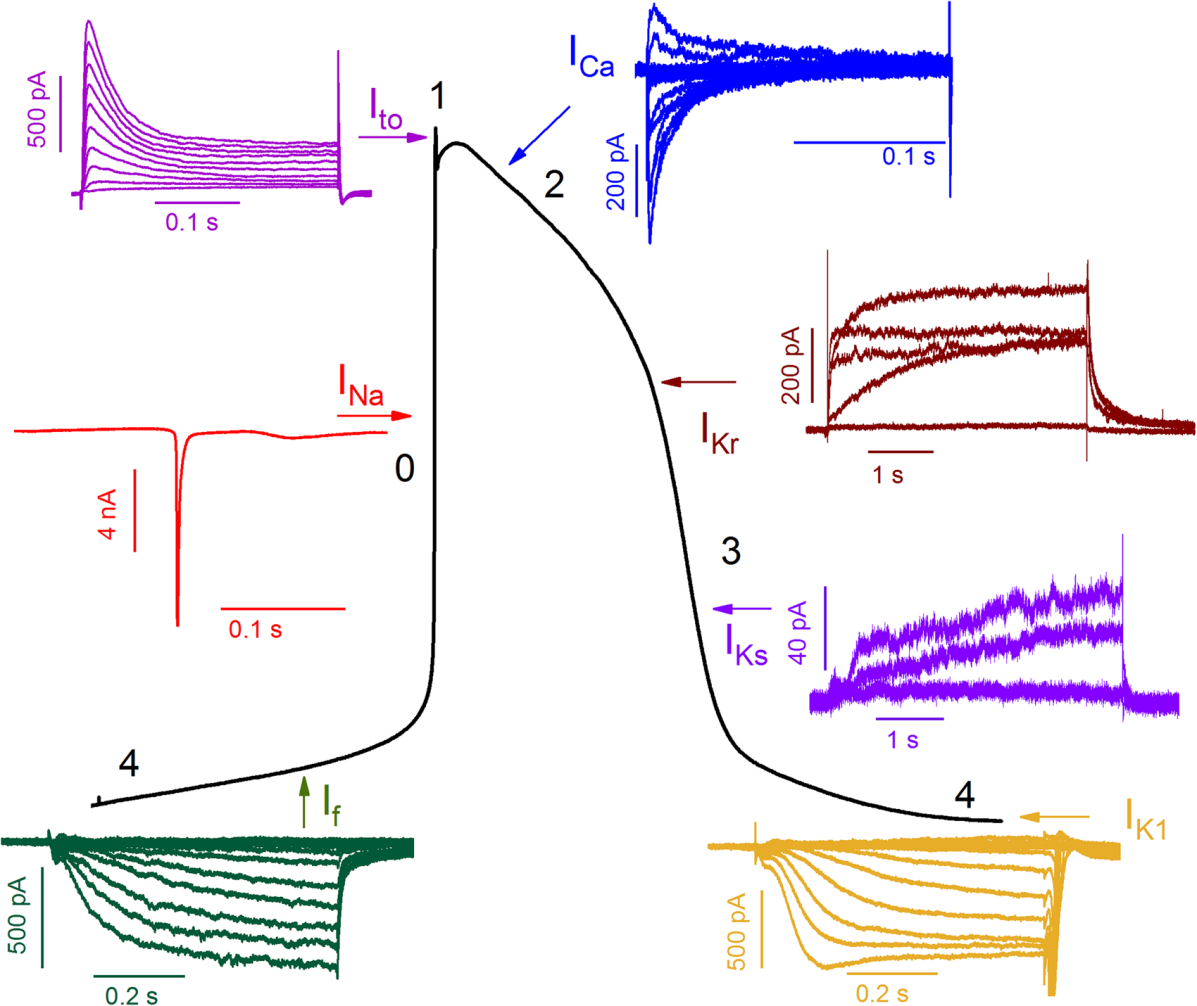
**Representative voltage-gated ionic current traces from hiPSC-CMs and their predominance area in action potential.** I_to_, transient outward potassium current; I_Ca_, calcium current; I_Na_, sodium current; I_Kr_/I_Ks_, rapid/slow rectifier potassium current; I_f_, funny current; I_K1_, inward rectifier potassium current. See Fig. S1 for current-voltage relationship of respective ionic currents.

The presence of various voltage-gated ionic currents in hiPSC-CMs were confirmed using voltage clamp techniques. Fig. 2 summarizes the representative AP and different ionic currents recorded from hiPSC-CMs. The fast I_Na_ is responsible for the rapid upstroke phase in AP, and peak I_Na_ current density was −23.1±5.2 pA/pF (*n*=15; Fig. S1A). Following rapid depolarization, transient outward potassium current (I_to_) starts the repolarization of AP. We found that the peak I_to_ current density at 70 mV was 7.3±1.4 pA/pF (*n*=5; Fig. S1B). The calcium current (I_Ca_) is responsible for the plateau phase of AP, and peak I_Ca_ current density at 10 mV was 2.6±0.7 pA/pF (*n*=5; Fig. S1C). As the plateau phase moves toward more negative membrane potential, mainly two types of potassium currents, rapid rectifier potassium current (I_Kr_) and slow rectifier potassium current (I_Ks_), are activated. I_Kr_ peak current was calculated at the end of 3 s depolarization test potential and I_Kr_ tail current is calculated as peak current in response to step depolarization. The peak and tail current densities of I_Kr_ at 0 mV were 1.0±0.3 and 1.1±0.2 pA/pF, respectively (*n*=5; Fig. S1D). Similarly, the peak and tail current densities of I_Ks_ at 40 mV were 0.6±0.1 and 0.5±0.1 pA/pF, respectively (*n*=6; Fig. S1E). In addition, inward rectifying potassium current (I_K1_) is activated during and following the repolarization phase to ensure the terminal repolarization and stable resting membrane potential. The peak I_K1_ current density and funny current (I_f_) density at 130 mV were −2.5±0.6 pA/pF and −3.7±1.0 pA/pF, respectively (*n*=6, Fig. S1F-G).

### Correlation of CaT with AP

**Fig. 3.**
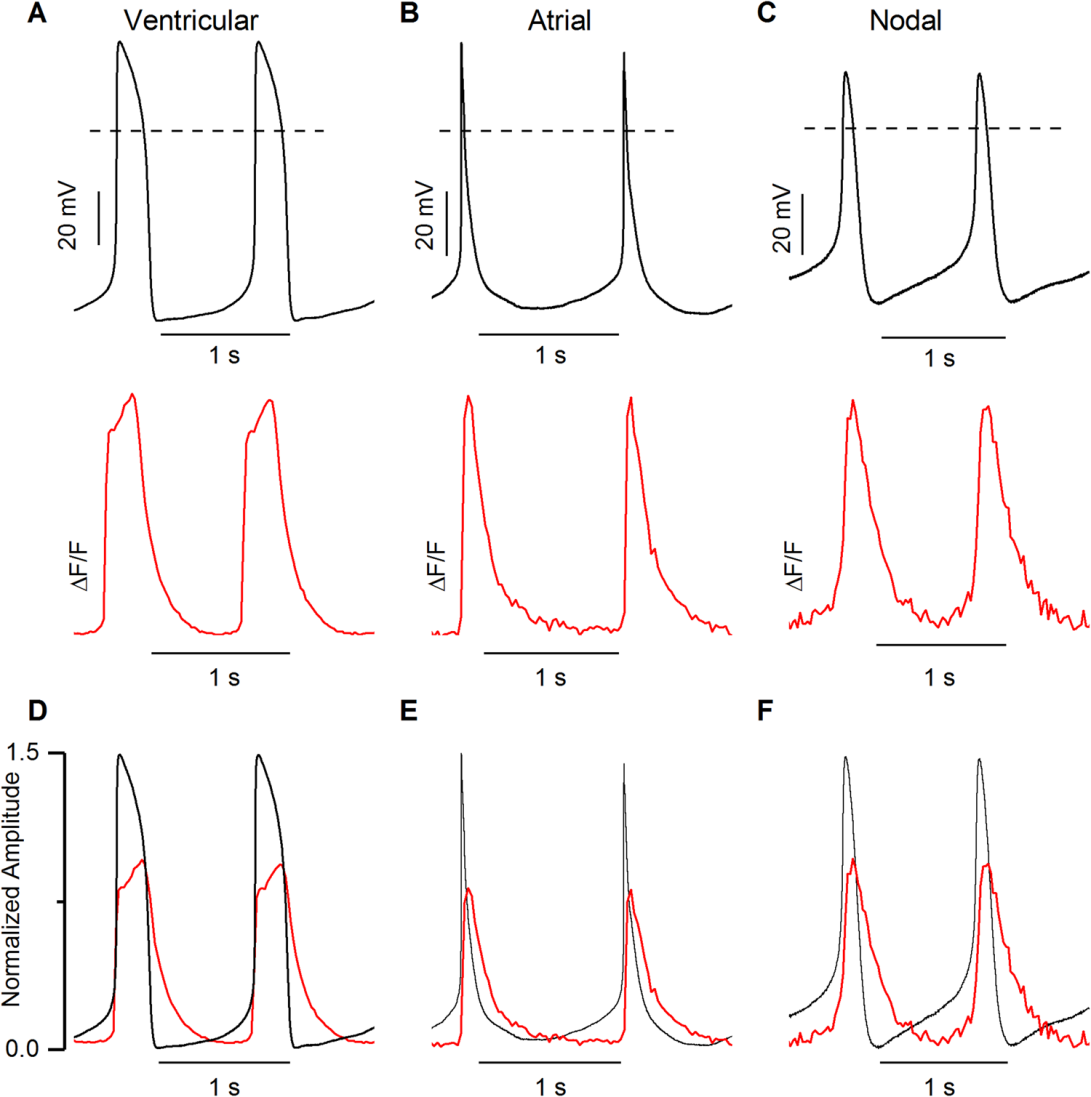
**Simultaneous recording of action potentials and intracellular calcium recording in hiPSC-CMs.** (A-C) Representative traces of action potentials (black, upper traces) and corresponding CaTs (red, lower traces) recorded simultaneously in (A) ventricular-like, (B) atrial-like and (C) nodal-like hiPSC-CMs. Dashed lines represent 0 mV. (D-F) Action potential amplitudes and CaT amplitudes were normalized to 1.5 and 1 value respectively. Ventricular-like hiPSC-CMs, *N*=37; atrial-like hiPSC-CMs, *N*=2; nodal-like hiPSC-CMs, *N*=2.

**Fig. 4.**
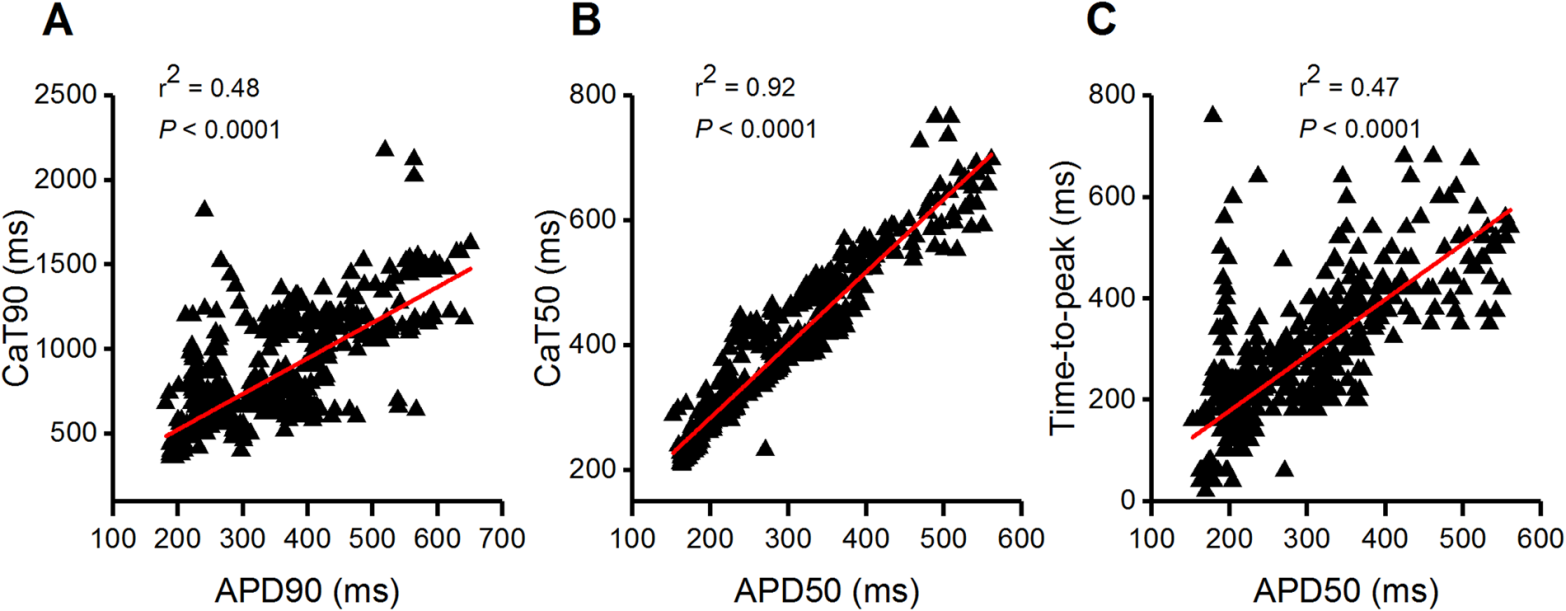
**Correlation between action potential and calcium transient parameters in ventricular-like hiPSC-CMs.** (A-C) The linear relationship between (A) APD90 and CaT90, (B) APD50 and CaT50 and (C) APD50 and time-to-peak. *N*=37, *n*=583, *P*<0.0001, Pearson’s correlation test. AP parameters were compared with their corresponding CaT parameters.

Spontaneously beating hiPSC-CMs with visible Ca2^+^ dynamics under a microscope were chosen for simultaneous recording of APs and CaTs. APs were continuously recorded in gap-free mode, while CaTs were recorded in between for a minimum of 30 s. The majority of the patched cells (∼90%) were characterized as ventricular-like hiPSC-CMs. Only a minority of the patched cells were atrial-like (∼5%) or nodal-like (∼5%) hiPSC-CMs. Examples of APs and CaTs recorded from the same cells representing all three types of hiPSC-CMs are shown in Fig. 3. As shown in Fig. 3, the upstroke of AP was followed by a rapid increase in [Ca^2+^]_i_. Notably, the [Ca^2+^]_i_ decreased to the minimum diastolic level after the repolarization phase of AP in hiPSC-CMs subtypes. Thus, the CaT at 90% decay (CaT90) was always longer than AP duration (APD) at 90% repolarization (APD90) irrespective of hiPSC-CMs subtypes. Our results showed that the average APD90 and CaT90 in ventricular-like hiPSC-CMs were 326.3±4.5 ms (*N*=37, *n*=583) and 790.7±13.7 ms (*N*=37, *n*=583), respectively. This implies that CaT90s were approximately 2.4 times longer than APD90s (Fig. 3A,D). The average APD90 and CaT90 in atrial-like hiPSC-CMs were 235.5±14 ms (*N*=2, *n*=35) and 552.8±18.3 ms (*N*=2, *n*=35), respectively, and thus CaT90s were approximately 2.3 times longer than APD90s (Fig. 3B,E). The same was observed with nodal-like hiPSC-CMs, the average APD90 and CaT90 were 195.4±3.0 ms (*N*=2, *n*=49) and 512.5±12.4 ms (*N*=2, *n*=49), respectively, therefore CaT90s were 2.6 times longer than APD90s (Fig. 3C,F). We performed the correlation tests between APD90 and CaT90; APD at 50% repolarization (APD50) and CaT at 50% decay (CaT50) and APD50 and time-to-peak of CaT in ventricular-like hiPSC-CMs (Fig. 4). Our results demonstrated a positive correlation (*N*=37, *n*=583, *P*<0.0001, Pearson's correlation test) between these parameters, indicating that CaT parameters and AP parameters are interdependent. The END-2 differentiation technique produces a lower number of atrial-like and nodal-like hiPSC-CMs, thus only small number of those cell types were recorded in this study. Therefore, we did not perform correlation tests in atrial- and nodal-like hiPSC-CMs.

### AP and CaT during arrhythmias

**Fig. 5.**
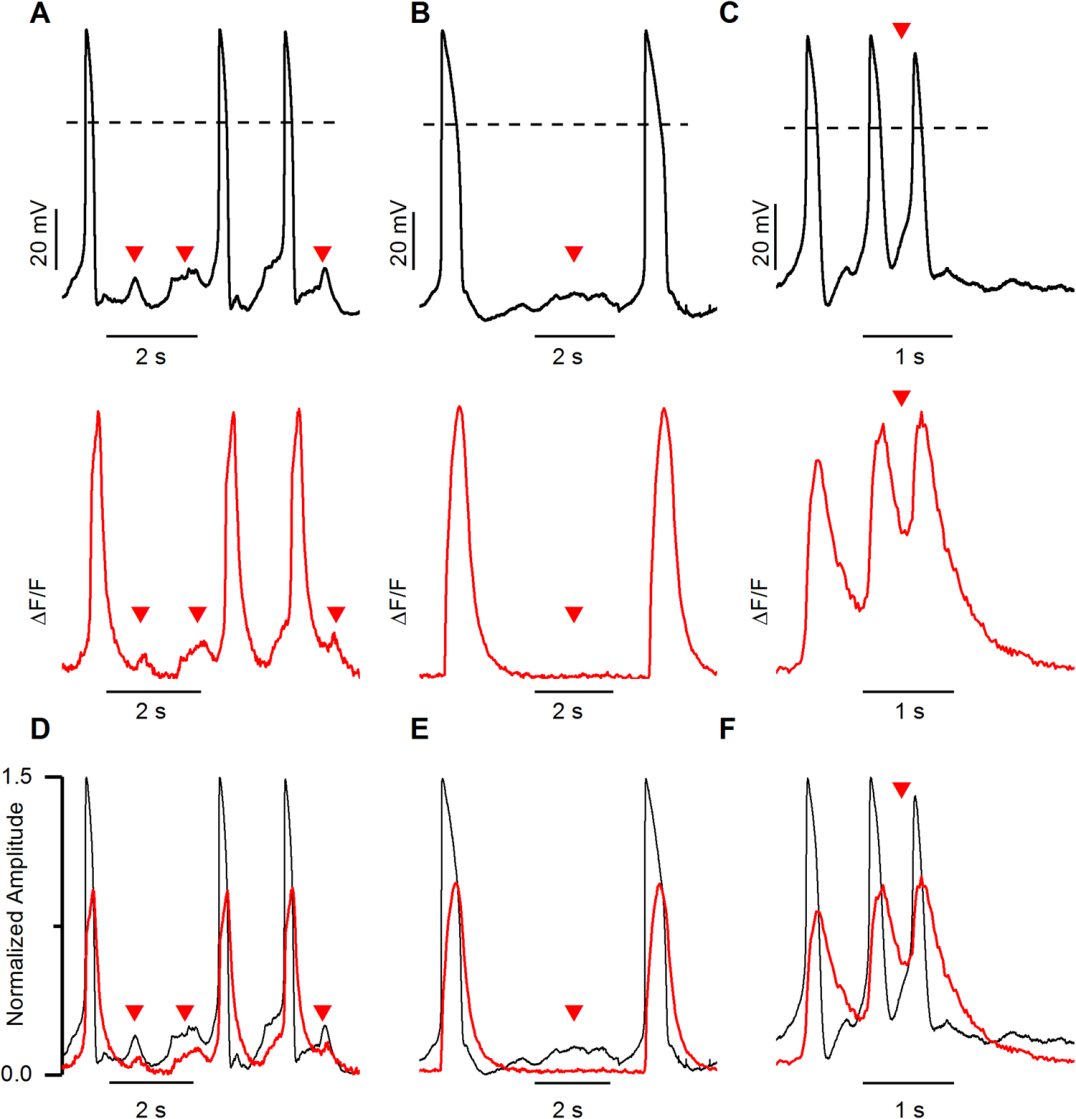
**Membrane potentials and the corresponding calcium transient during arrhythmia in hiPSC-CMs.** (A) Action potentials exhibiting DADs (black, upper trace) with corresponding elevation in CaT (red, lower trace). (B) Action potentials exhibiting DADs (black, upper trace) without corresponding elevation in CaT (red, lower trace). For more detail, see Fig. S2. (C) Action potentials exhibiting phase 3 EAD (black, upper trace) and corresponding CaT (red, lower trace). Note the elevation in intracellular calcium level in phase 3 EAD. (D-F) Action potential amplitudes and CaT amplitudes were normalized to 1.5 and 1 value respectively. Dashed lines represent 0 mV. Red arrowheads indicate the DADs and phase 3 EADs.

During simultaneous recording under baseline conditions, delayed after depolarizations (DADs) were occasionally observed (Fig. 5). DADs were defined as abnormal membrane depolarizations with amplitudes of ≥3% of the preceding AP that occurred after completion of the repolarization. Similarly, early after depolarizations (EADs) were defined as abnormal firings either during phase 2 or phase 3 of AP. At baseline, each DAD and phase 3 EAD recorded in AP were compared to their corresponding CaT of the same cell. Interestingly, 33 DADs in the V_m_ recording corresponded to [Ca^2+^]_i_ elevation (Fig. 5A,D). However, 22 DADs presented changes in V_m_ without any corresponding change in [Ca^2+^]_i_ i.e. DAD was observed in AP recording, but no change was observed in [Ca^2+^]_i_ (Fig. 5B,E; Fig. S2A). Furthermore, we performed a correlation test (Fig. S2A) on those 33 DADs with similar observations in V_m_ and CaT between relative DAD amplitude (i.e. % of DAD amplitude with respect to APA) and the corresponding relative CaT amplitude (i.e. % amplitude of [Ca^2+^]_i_ elevation with respect to CaT amplitude). The results demonstrated a positive correlation between these two parameters (*P*=0.01, Pearson's correlation test), implying that the amplitude of DADs was dependent on the amplitude of elevated [Ca^2+^]_i_. Also, we compared the amplitude of DADs with and without corresponding elevation in [Ca^2+^]_i_ (Fig. S2B). We found that the average DAD amplitude (10.4±0.6 mV; *n*=33) with corresponding elevation in [Ca^2+^]_i_ was significantly higher than the average DAD amplitude (6.5±0.5 mV; *n*=26) without corresponding [Ca^2+^]_i_ elevation (*P*<0.0001*,* student *t*-test). In addition, phase 3 EADs were recorded during the simultaneous recording of V_m_ and CaT during baseline conditions (*n*=2, Fig. 5C,F) and elevated [Ca^2+^]_i_ was observed in CaT at the same time as in phase 3 EADs observed in V_m_.

### Effect of ion channel blockers on AP and CaT

**Fig. 6.**
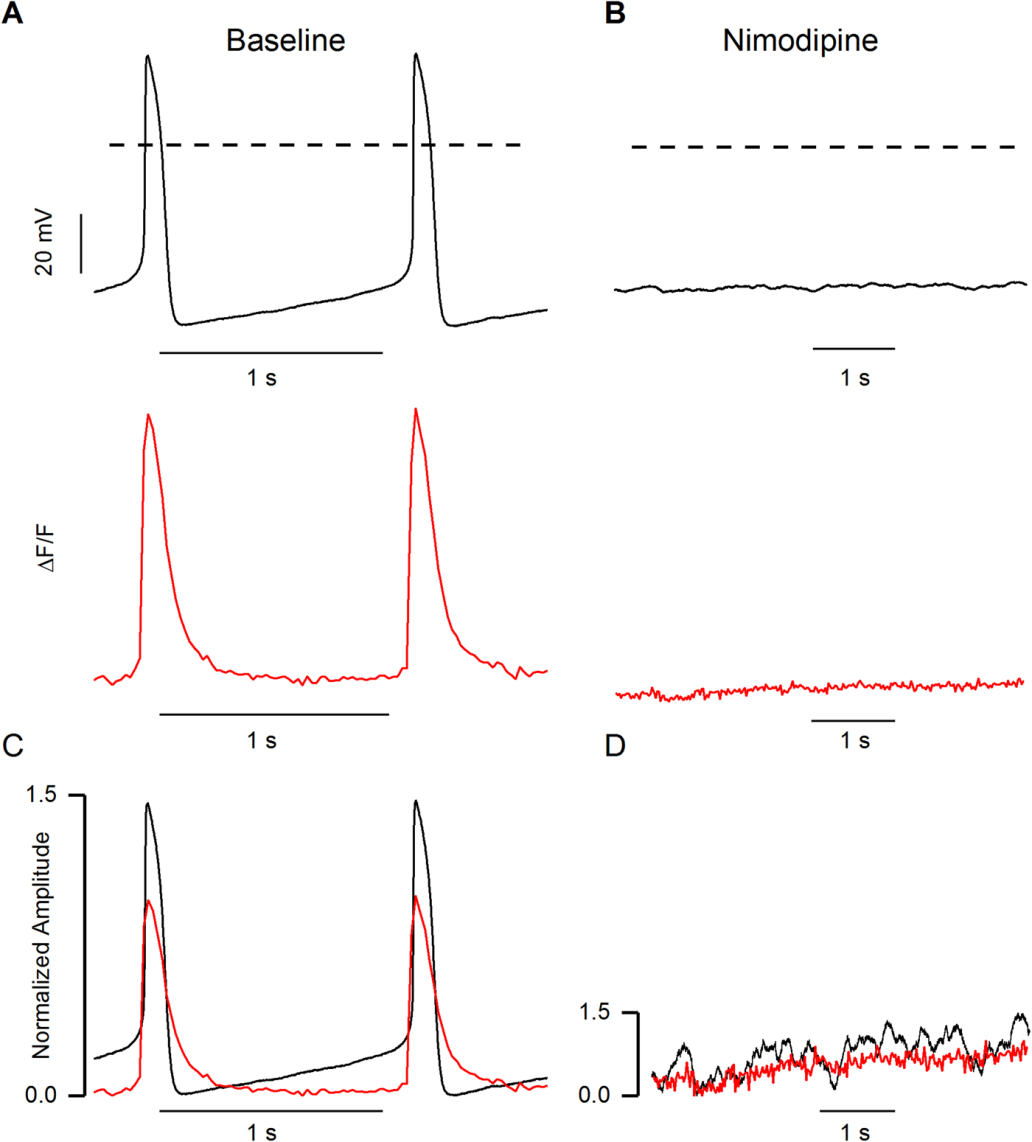
**Effect of 5 µM Nimodipine in spontaneous beating of hiPSC-CMs.** (A) Baseline recording of action potentials (black, upper trace) and corresponding CaT traces (red, lower trace) from the same hiPSC-CMs. (B) Representative traces of membrane potential (black, upper trace) and CaT (red, lower trace) showing the cessation of spontaneous beating with blockage of calcium channels. (C,D) Action potential amplitudes and CaT amplitudes were normalized to 1.5 and 1 value respectively. Dashed lines represent the 0 mV. (*N*=3).

**Fig. 7.**
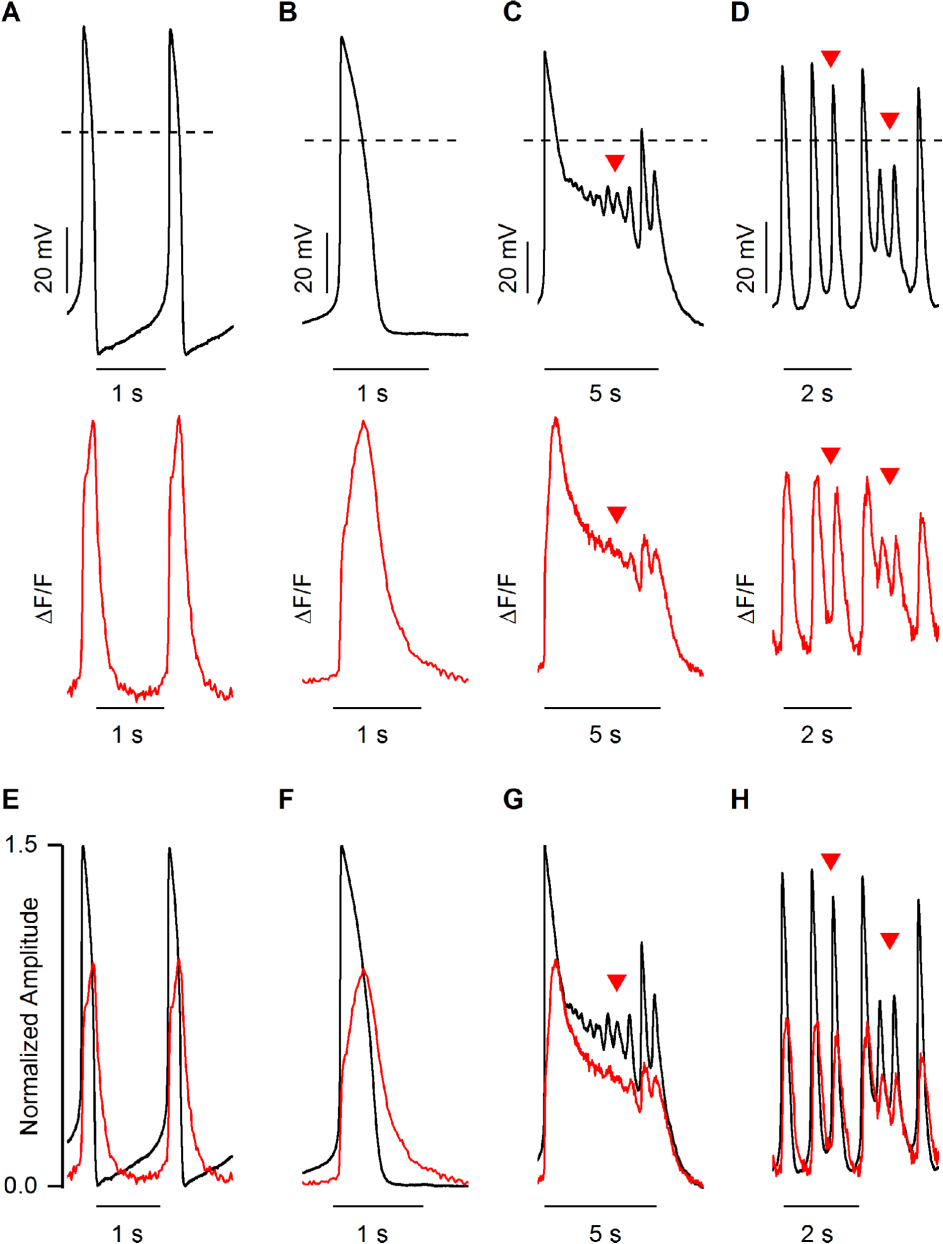
**Three different responses of 650 nM E-4031 in spontaneous beating of hiPSC-CMs.** (A) Representative traces of baseline action potential (black, upper trace) and corresponding CaT (red, lower trace). (B) Prolongation of action potential duration (black, upper trace) and corresponding CaT (red, lower trace). (C) Occurrence of phase 2 EAD (black, upper trace) and corresponding CaT (red, lower trace). (D) Oscillation of membrane potential with EADs (black, upper trace) corresponding with CaT (red, lower trace). (E-H) Action potential amplitudes and CaT amplitudes were normalized to 1.5 and 1 value respectively. Dashed lines represent the 0 mV. Red arrowheads indicate the phase 2 and phase 3 EADs.

Initially, simultaneous APs and CaTs were recorded in a normal extracellular solution. To test whether I_Ca,L_ is important for the spontaneous beating of hiPSC-CMs, 5 µM nimodipine was used (Fig. 6A). This caused the cessation of both AP and CaT in all of the cells tested (*N*=3, Fig. 6B). E-4031 (650 nM) was used to block the I_Kr_ in hiPSC-CMs, and after 1 min of exposure to E4031, four types of responses were observed (Fig. 7). Firstly, 10% (*N*=1/10) of cells showed prolongation of both APD and CaT, but there was no occurrence of EAD (Fig. 7A,D). The maximum diastolic potential (MDP) was decreased to −50 mV and the APD90/50 ratio became 1.4±0.01 (*n*=12), meaning APs became slightly more triangular. In addition, the APD90 and CaT90 were 420.9±3.6 (*n*=12) and 891.7±8.7 (*n*=12) respectively; increasing by 72% and 77%, respectively, from their baseline values. Secondly, 40% (*N*=4/10) of cells showed prolongation of both APD and CaT duration and this eventually led to phase 2 EADs (Fig. 7B,E). The CaT90s were extended in proportion to the prolongation of APD90s and CaT followed the shape of V_m_. In EADs, the average APD90 and CaT90 were 2334.3±508.2 ms and 2471.1±508.7 ms (*n*=27), respectively. Notably, the CaT90 was approximately 1.1 times longer than the APD90. Thus, the gaps between V_m_ and [Ca^2+^]_i_ were closer during the terminal repolarization in phase 2 EAD episodes. Thirdly, 30% (*N*=3/10) of cells had depolarized MDP and increased in beating frequency, i.e. an oscillation configuration with or without the occurrence of phase 2 and phase 3 EADs (Fig. 7C,F). The MDP was decreased to 42.7±3.4% (*N*=3) and beating frequency was increased by 110.6±43.1% (*N*=3). In these cases, the APD90s were increased by 41% and the average value was 355.2±3.7 (*n*=60). In contrast, the CaT90s were decreased by 2.4% and the average value was 547.3±6.9 (*n*=60). Although CaT90s were decreased from baseline, APD90s were still shorter than CaT90 in oscillation conditions. Moreover, the APD90/APD50 ratio was increased to 1.8±0.01 (*n*=60), indicating that AP shape became more triangular than in baseline conditions. Finally, 20% (*N*=2/10) of cells showed a cessation of beating, with minimal V_m_ and [Ca^2+^]_i_ fluctuation (data not shown).

## DISCUSSION

In this study, we found that, in hiPSC-CMs: (1) voltage-gated ionic currents are functional; (2) there are strong correlations between AP parameters and CaT parameters; (3) DADs observed in V_m_ recordings are mostly characterized by elevation in [Ca^2+^]_i_; (4) blocking of I_Kr_ causes variable responses, including prolongation of both APD and CaT duration, occurrence of EAD and even cessation of beating. The AP recordings from hiPSC-CMs exhibit all phases of AP and current densities of different voltage-gated channels measured in the present study are comparable to earlier studies in hiPSC-CMs (Han et al., 2014; Spencer et al., 2014; Lee et al., 2016; Ma et al., 2015). The depolarization of V_m_ causes the opening of I_Ca,L_, and facilitates the main pathway for Ca^2+^ entry for CICR (Kane et al., 2015). However, in this study, blocking the I_Ca,L_ resulted in spontaneous beating ending, which is consistent with earlier studies (Itzhaki et al., 2011; Spencer et al., 2014), indicating the importance of I_Ca,L_ in the normal function of CMs. The Ca^2+^-activated transient currents influence the characteristics of normal AP (Laflamme and Becker, 1996). Similarly, abnormal intracellular Ca^2+^ handling cause the dysfunction of contraction/relaxation and arrhythmia in diseased CMs (Kujala et al., 2012; Penttinen et al., 2015; Ojala et al., 2016).

This study demonstrated that in normal conditions, the CaT90s were twice as long in duration than APD90s in hiPSC-CMs. In an earlier study of simultaneous voltage and Ca^2+^ mapping using Fura-4F in the hiPSC-CMs monolayer, the CaT90s were also observed to be longer than APD90s (approximately 1.2 times) (Lee et al., 2012). A possible reason for the larger difference in our study compared to earlier studies might be due to the Ca^2+^ indicators: there was low-affinity ratiometric Fura-4F in previous studies as opposed to high-affinity non-ratiometric Fluo-4 as Ca^2+^ dye in our study. Ca^2+^ dye with high-affinity (Fluo-4) had significantly longer CaT duration than Ca^2+^ dye with low-affinity (Fura-4F) (Kong and Fast, 2014; Fast et al., 2004).

Our earlier studies (Ojala et al., 2016; Kujala et al., 2012; Prajapati et al., 2018) and studies by other groups (Itzhaki et al., 2012; Liang et al., 2013; Lan et al., 2013) have shown that control hiPSC-CMs also occasionally exhibited arrhythmias (DADs and phase 3 EADs) under baseline conditions. However, phase 2 EADs were not observed in control hiPSC-CMs at baseline, but were commonly observed in disease conditions such as LQT1 and LQT2 (Kuusela et al., 2017; Spencer et al., 2014; Ma et al., 2015). In addition, we demonstrated that DADs and EADs could take place in the same CMs (Ojala et al., 2016; Prajapati et al., 2018) particularly because spontaneous SR Ca^2+^ release plays a role in the occurrence of both DADs and EADs (Volders et al., 1997). The well-established mechanism of DADs is that the spontaneous release of Ca^2+^ from SR induces transient inward current generated either by the activation of NCX, calcium-activated Cl^−^ current or non-selective cationic current. (Ko et al., 2017; Verkerk et al., 2000; Schlotthauer and Bers, 2000). A similar DAD mechanism was also observed in this study with hiPSC-CMs where elevation in [Ca^2+^]_i_ resulted in the rise in V_m_. However, we also observed events where [Ca^2+^]_i_ did not change, although DADs were observed in V_m_. We postulate three possible explanations for this discrepancy: (1) the [Ca^2+^]_i_ amplitude got reduced by spatial averaging since the amplitude of DADs without [Ca^2+^]_i_ elevation was significantly lower, (2) high-affinity Fluo-4 artificially prolongs the CaT duration, which overshadowed the DAD observed nearer to terminal repolarization of AP, or (3) the involvement of Ca^2+^-independent currents promoting depolarization of V_m_, and possible mechanisms is still unknown. Consistent with a previous study (Mitsunori et al., 2009), the amplitude of DAD was dependent on the amplitude of [Ca^2+^]_i_ elevation in our study. Furthermore, the DAD amplitude is also dependent on the sensitivity of resting V_m_ to change in [Ca^2+^]_i_ i.e. diastolic Ca^2+^-voltage coupling gain (Mitsunori et al., 2009). On the other hand, APDs and CaTs were prolonged concurrently with the pharmacological blockage of I_Kr_ by E-4031; phase 2 EADs were also occasionally observed. During phase 2 EAD, the notable observation was that the CaT always followed the V_m_ during the EAD episodes i.e. CaT was changing corresponding to small V_m_ oscillations. This demonstrates a strong dependency between V_m_ and CaT during this kind of arrhythmic condition. Furthermore, the upstroke of an EAD is generally carried by I_Ca_, and take-off potential of an EAD depends on the complex interplay between the kinetics of I_Ca_ and I_Ks_ (Chang et al., 2011). The mechanism of phase 2 EAD is that the window current of I_Ca_ overlapping the V_m_ promotes the reactivation of I_Ca,L_ and causes EAD to occur (January and Riddle 1989). In addition, an earlier study explained that spontaneous SR Ca^2+^ release during the plateau phase of AP has an essential role in the development of an EAD (Choi et al., 2002). The phase 3 EAD shares the properties of both DADs and phase 2 EADs, but it has its own unique character. Phase 3 is distinguished by the breaking off in the final phase of repolarization of AP. The elevated [Ca^2+^]_i_ during repolarization enhances the NCX current that could potentially trigger phase 3 EADs (Volders et al., 2000; Maruyama et al., 2012). Our study demonstrates that the CaT was also following the change in V_m_ during the phase 3 EAD, similarly as in the phase 2 EAD. An earlier study using Langendorff rabbit heart reported that the CaT faithfully tracts the V_m_ during faster beating frequency and ventricular tachycardia (Wu et al., 2005). However, there is a possibility that SR Ca^2+^ cycling undergoes an intrinsic dynamic independent of V_m_. One example of such is during ventricular fibrillation (VF), where CaT is no longer associated with V_m_ (Omichi et al., 2004; Wu et al., 2005). Taken together, CaT and V_m_ are closely associated not only in normal condition, but also in phase 2/3 EADs and in the majority of DADs. However, CaT can dissociate from V_m_, and go through its own pathway in certain conditions, such as in a VF episode.

## MATERIALS AND METHODS

### Ethical approval and generation of hiPSC lines

Approval from the Ethics Committee of Pirkanmaa Hospital District was given to conduct the research on hiPSC lines (Aalto-Setälä R08070). Patients donating skin biopsies gave informed consent in Tays Heart Hospital, Tampere University Hospital, Finland. Two control hiPSC lines, UTA.04602.WT (healthy 56-year-old female) and UTA.04511.WT (healthy 34-year-old male) were used in this study. The UTA.04511.WT hiPSC line was generated using Sendai vectors, and UTA.04602.WT was generated by using pMX retroviral vectors without Cre-LoxP site. Both hiPSC lines were derived and cultured on mouse embryonic fibroblast (MEF) feeder cell layers (26,000cells/cm2; CellSystems Biotechnologie Vertrieb GmbH, Troisdorf, Germany) in culture medium containing knockout-DMEM (ko-DMEM) (Gibco) supplemented with 20% knockout serum replacement (ko-SR) (Gibco), 1% nonessential amino acids (NEAA) (Lonza Group Ltd, Basel, Switzerland), 2 mM GlutaMax (Gibco), 50 U/ml penicillin/streptomycin (Lonza Group Ltd), 0.1 mM 2-mercaptoethanol (Gibco) and 4 ng/ml basic fibroblast growth factor (bFGF) (PeproTech, Rocky Hill, USA). The characterization of these lines were found in previous study (Ojala et al., 2016).

### Differentiation into cardiomyocytes and dissociation

Both hiPSC lines were differentiated into cardiomyocytes by co-culturing with Mitomycin C (Sigma-Aldrich) treated mouse visceral endodermal-like cells (END-2) (Hubrecht Institute, Utrecht, Netherlands) (50,000cells/cm^2^) as described earlier (Mummery et al., 2003; Ojala et al., 2012). MEF feeder cell layers were removed manually before differentiation. Approximately 30 colonies per well were detached and transferred onto END-2 cells in stem cell culture medium without ko-SR or bFGF and supplemented with 3 mg/ml ascorbic acid (Sigma-Aldrich). Medium was changed after 5, 8 and 12 days of culturing. After 15 days of culturing, 10% ko-SR was included and ascorbic acid was excluded from the culture medium; subsequently, the medium was changed three times per week. Beating areas were cut and washed in Low-Ca buffer [12 ml 1 M NaCl, 0.54 ml 1 M KCl, 0.5 ml 1 M MgSO4, 0.5 ml 1 M Na pyruvate, 2 ml 1 M glucose, 20 ml 0.1 M taurine and 1 ml 1 M HEPES (pH adjusted to 6.9 with NaOH)] at room temperature (RT) for 30 min. Beating areas were dissociated in buffer including 1 mg/ml collagenase A (Roche Diagnostics, Basel, Switzerland) at 37°C for 45 min [12 ml 1 M NaCl, 3 µl 1 M CaCl2, 0.54 ml 1 M KCl, 0.5 ml 1 M MgSO4, 0.5 ml 1 M Na pyruvate, 2 ml 1 M glucose, 20 ml 0.1 M taurine and 1 ml 1 M HEPES (pH adjusted to 6.9 with NaOH) (all from Sigma Aldrich)] and washed in KB medium at RT for 1 hour (3 ml 1 M K2HPO4, 8.5 ml 1 M KCl, 2 mmol/l Na2ATP, 0.5 ml 1 M MgSO4, 0.1 ml 1 M EGTA, 0.5 ml 1 M Na pyruvate, 2 ml 1 M glucose, 5 ml 0.1 M creatine and 20 ml 0.1 M taurine, pH 7.2) (all from Sigma Aldrich) (20 µl of 1 M glucose was added per 1 ml of KB-medium before use). Single cells were plated onto 0.1% gelatin-coated glass coverslips in EB medium, consisting of ko-DMEM supplemented with 20% fetal bovine serum (FBS) (Biosera, Boussens, France), 1% NEAA, 2 mM GlutaMax and 50 U/ml penicillin/streptomycin. In this study, 40–60 day-old hiPSC-CMs were used.

### Immunocytochemistry

Cardiomyocytes were dissociated onto 12 mm diameter coverslips. After 7 days, CMs were fixed with 4% paraformaldehyde (Sigma-Aldrich) and stained with cTnT (1:500; Abcam), I_Ca,L_ (Ca_V_1.2, 1:500; Alomone labs, Jerusalem, Israel) and RyR2 (1:200; Alomone labs) primary antibodies. The secondary antibodies were Alexa-Fluor-488-anti-goat-IgG, Alexa-Fluor-568-anti-rabbit-IgG (1:800) (all three from Thermo Fisher Scientific). Cells were mounted with Vectashield (Vector Laboratories, Burlingame, USA) containing DAPI for staining nuclei. The z-stack images were captured with Nikon A1 confocal laser-scanning microscope (Nikon, Tokyo, Japan) using 60× oil immersion objectives (N.A.=1.4, Nikon). The images were further processed using ImageJ (1.51 g; NIH) and Adobe Photoshop CC 2017.

### Chemicals

All the drugs and chemicals for the experiments were purchased from Sigma-Aldrich unless otherwise specified. Potassium methanesulfonate (KMeSO_4_) was ordered from MP Biomedicals (California, USA). Stock solutions for extracellular solution were prepared in 1 M concentration and refrigerated until use. Extracellular solution was made freshly by diluting stock solution (in mM): 143 NaCl, 4.8 KCl, 1.8 CaCl_2_, 1.2 MgCl_2_, 5 glucose and 10 HEPES (pH adjusted to 7.4 with NaOH). Intracellular solution was made and refrigerated (in mM): 132 KMeSO_4_, 4 EGTA, 20 KCl, 1 MgCl_2_ and 1 CaCl_2_ (pH adjusted to 7.2 with KOH). E-4031, nimodipine and chromanol 293B were dissolved in Milli-Q™ water (Millipore), ethanol (EtOH) and dimethyl sulfoxide (DMSO) respectively, and stored frozen. Cadmium chloride (CdCl_2_) and barium chloride (BaCl_2_) were dissolved in Milli-Q™ water and stored at +4°C. On the day of the experiments, the drugs were diluted to the final concentration in extracellular solution. 4-Aminopyridine (4-AP) was dissolved in the extracellular solution on the day of experiment. Amphotericin-B was first dissolved in DMSO, and this solution was added to intracellular solution to make final concentration of 0.24 mg/ml.

### Simultaneous patch clamp and calcium imaging

Cells on a coverslip were loaded with 4 μM Fluo-4 AM (Thermo Fisher Scientific) for 30 min in extracellular solution at 37°C. Extracellular solution was heated to 35-36°C with inline heater SH-27B controlled with a TC-324B controller unit (Warner Instruments Inc., Hamden, USA). The perfusion was controlled by a gravity driven VC38 application system (ALA Scientific Instruments Inc., New York, USA). Patch pipettes (Harvard Apparatus Ltd, Holliston, USA) were freshly prepared using PC-10 micropipette pullers and flame polished with MF-830 microforge (both from Narishige Int., Tokyo, Japan). The patch electrodes had tip resistance of 2.0-3.0 MΩ with intracellular solution. AP recordings and Ca^2+^ imaging were performed simultaneously. For patch clamp experiment, perforated patch using Amphotericin B was performed to record AP in Axon Series 200B patch-clamp amplifier connected to Digidata 1440a AD/DA converter driven by pCLAMP 10.2 software (all from Molecular Devices LLC, San Jose, USA). APs were recorded in gap-free mode in the current-clamp from the spontaneously beating hiPSC-CMs. Current-clamp recordings were digitally sampled at 20 kHz and filtered at 2 kHz using a low pass Bessel filter on the recording amplifier. Ca^2+^ imaging was performed using ANDOR iXon 885 EM-CCD camera (Andor Technology, Belfast, Northern Ireland) synchronized with a Polychrome V light source controlled by a real time control unit. Ca^2+^ kinetics of spontaneously beating CMs were imaged with an inverted Olympus IX70 microscope using UApo/340 0.75NA 20× air objective (Olympus, Tokyo, Japan) and recorded with LiveAcquisition software (TILL Photonics, Munich, Germany), which was used to control light source and camera during recording. Fluo-4 was excited at 490 nm wavelength and the emission was recorded through Olympus U-MF2 Alexa 488 band-pass filter cube (excitation 470-495, emission 525/50 nm). The sampling interval was 20 ms. The pCLAMP software was configured to perform the synchronization between patch clamp and Ca^2+^ imaging system. When the Ca^2+^ imaging started, the synchronization pulse (SP) was sent from the imaging system to patch clamp system, thus SPs were recorded alongside with APs, and the SP stopped once the Ca^2+^ imaging stopped recording. The schematic diagram and video illustrating simultaneous recordings of patch clamp and CaT is shown in Fig. S3 and Movie 1.

### Voltage clamp

All the voltage clamp experiments were performed at 35-36°C. The extracellular and intracellular solutions were similar to those used in the current clamp experiments, with the addition of specific ion channel blockers. The I_Na_ was measured with ramp protocol, with a holding potential (HP) of −80 mV. First, the cells were depolarized to −120 mV for 50 ms and then ramped up to 40 mV with rate of 0.53 V/s. The I_Ca_ were measured in the presence of 3 mM 4-AP to block I_to_, and an HP of −40 mV. To elicit the Ca^2+^ current, step-protocol from −60 mV to 70 mV with the step-size of 10 mV was used. The I_to_ was measured in the presence of 300 µM CaCl_2_ to block the Ca^2+^ current, and HP of −80 mV. Two-step protocol was used: first step was −50 mV for 50 ms to inactivate sodium channels, then to test potential of 500 ms duration from −50 mV to 70 mV with step-size of 10 mV. The I_Kr_ was measured as 1 µM E4031-sentitive current in the presence of 5 µM nimodipine and 10 µM chromanol to block I_Ca_ and I_Ks_ respectively. Using the HP of −40 mV, step protocol from −20 to 40 mV of 3 s with step size of 20 mV was used. The I_Ks_ was measured as 10 µM chromanol 293B-sentitive current in the presence of 5 µM nimodipine and 1 µM E-4031 to block I_Ca_ and I_Kr_ respectively. Using the HP of −40 mV, step protocol from 0 to 40 mV of 3 s with step-size of 20 mV was used. The I_K1_ and I_f_ were measured as 2 mM BaCl_2_ sensitive and insensitive current respectively in the presence of 300 µM CdCl_2_ and 3 mM 4-AP. From the holding potential of −40 mV, test potential from −140 mV to 0 mV with duration of 700 ms and step-size of 10 mV was used.

### Data analysis and statistics

Recorded APs were analyzed with OriginTM 9.1 (OriginLab Corp., Northampton, USA) to extract APD50 and APD90. Voltage clamp data were analyzed using Clampfit software version 10.5 (Molecular Devices LLC). For Ca^2+^ imaging analysis, a whole cell region of interest (ROI) was drawn. Acquired signal was normalized as ΔF/F_0_ using LiveAcquisition software (TILL Photonics). The Ca^2+^ traces were analyzed with Clampfit software to extract Ca^2+^ transient at 50% and 90% of repolarization (CaT50 and CaT90), and time-to-peak (time taken for CaT to reach its peak intensity). Each AP parameter was compared with its corresponding CaT parameter. Data from both hiPSC lines were combined. The hiPSC-CMs were categorized as ventricular-like and atrial-like CMs when they showed APD90/APD50<1.3 and APA>80mV; and APD90/APD50>1.35, respectively. Similarly, hiPSC-CMs were categorized as nodal-like CMs when they showed APD90/APD50>1.3 and APA<80mV with slower dV/dt. To check the correlation between two parameters, Pearson's correlation test was used. Differences between two groups were evaluated with student's unpaired *t*-test (two-tail). In this study, ‘*N*’ represents number of cells whereas ‘*n*’ represents number of AP or CaT used. *P*<0.05 was considered as statistically significant. Data is presented as mean±standard error of mean (s.e.m.).

## CONCLUSIONS

This study demonstrates the interrelation between CaT and V_m_ in hiPSC-CMs both during normal regular beating and in arrhythmic conditions. The simultaneous recording of V_m_ and CaT allows us to study the intracellular Ca^2+^ dynamics with respect to changes in V_m_ and vice versa. These experiments and models could be very helpful in understanding the correlation between V_m_ and CaT in more detail. Furthermore, hiPSC-CMs provide a safe and powerful tool to study the cardiac physiology and pathophysiology *in vitro*.

## Study limitation

Although hiPSC-CMs offer a robust platform for *in vitro* modeling of various genetic cardiac diseases, they have potential limitations because of their intrinsic differences compared to adult CMs. Some of the main ways that they do not fully resemble adult CMs are the lack of t-tubules and low expression of I_K1_. Furthermore, the majority of hiPSC-CMs differentiate into ventricular-like cardiomyocytes with our differentiation protocol, thus only limited atrial-like and nodal-like hiPSC-CMs are obtained. This hinders the ability to study the correlation between V_m_ and CaT in atrial and nodal cardiomycoytes. In addition, technical limitations exist in our measurement. During an AP measurement, the sampling rate was 20 kHz, which is enough to measure small changes in V_m_. However, the frame-recording interval of 20 ms in Ca^2+^ imaging is not fast enough to capture small intracellular dynamics, especially during phase 0 of AP.

## Supplementary Material

Supplementary information
