# Supplementary material for: Simultaneous recordings of action potentials and calcium transients from human induced pluripotent stem cell derived cardiomyocytes
Source: Biol Open. 2018 Jul 3;7(7):bio035030. doi: 10.1242/bio.035030 (PMC6078349; doi:10.1242/bio.035030)
Supplement: Supplementary information [file biolopen-7-035030-s1.pdf]

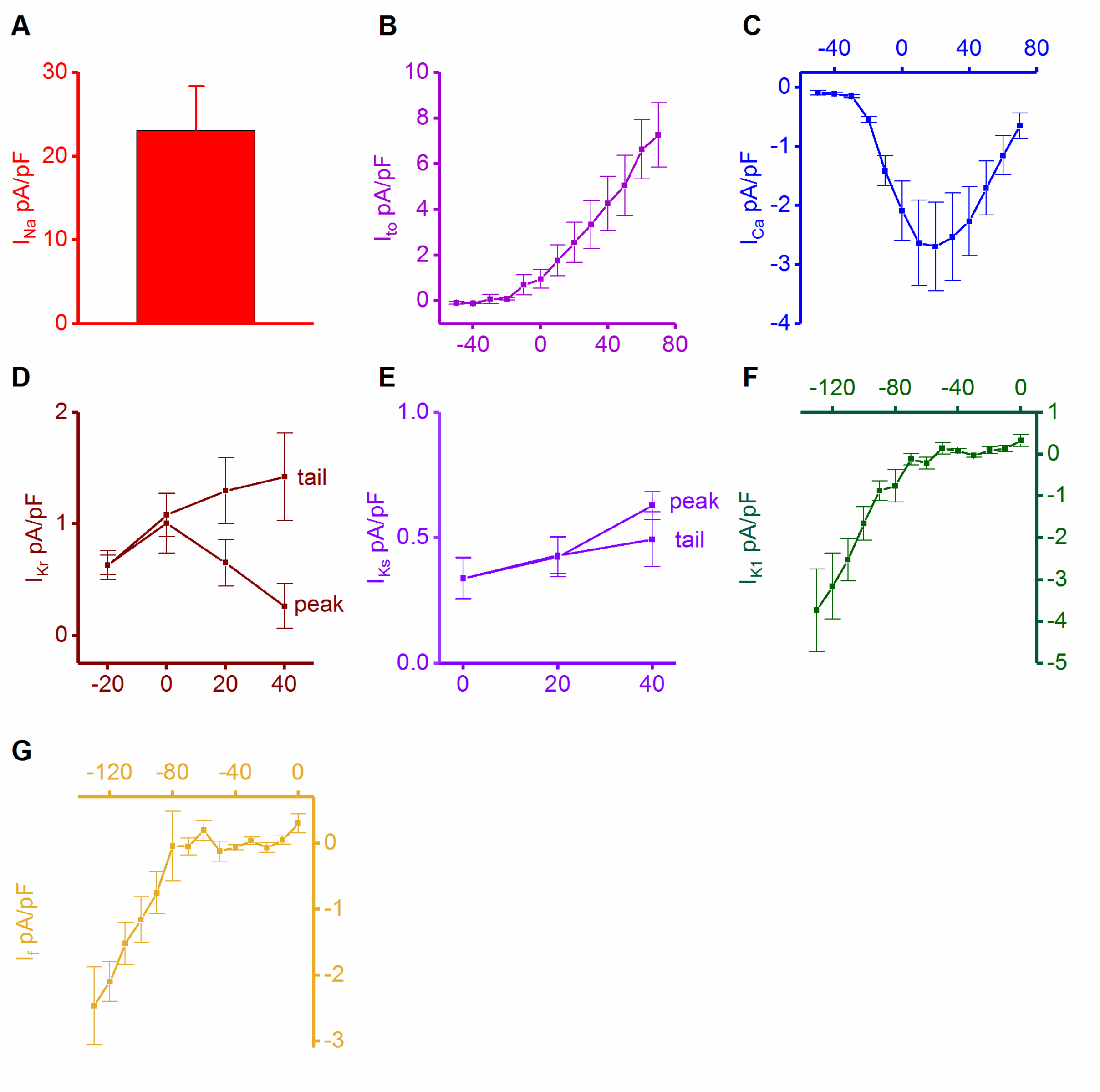

Figure S1: Current-voltage (I-V) relationship of different voltage-gated ionic channels. I-V curve of (A) sodium current (I_{Na}) density (B) transient outward potassium current (I_{to}) density (C) calcium current (I_{Ca}) density (D) rapid rectifier potassium current (I_{Kr}) density (E) slow rectifier potassium current (I_{Ks}) density (F) inward rectifier potassium current (I_{K1}) density (G) funny current (I_f) density.

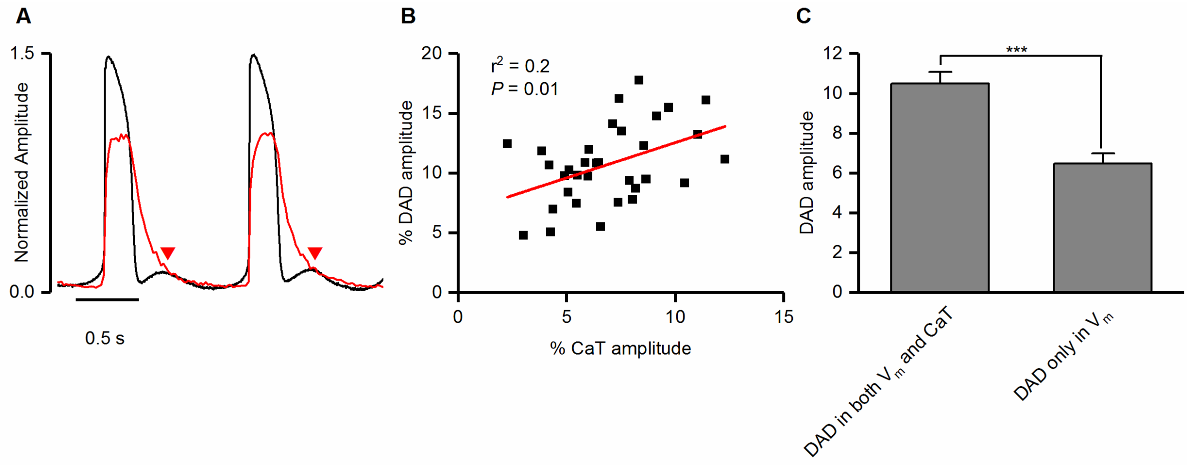

Figure S2: Characteristics of DADs in hiPSC-CMs. (A) Action potentials exhibiting DADs without corresponding change in CaT. (Action potential amplitudes and CaT amplitudes were normalized to 1.5 and 1 respectively) (B) The relationship between the percentage of DAD amplitude and percentage of CaT amplitude (r^2=0.2, P=0.01, Pearson's correlation test; n=33) (C) Average amplitude of DADs observed in both V_m and CaT (n=33), and only observed in V_m (n=26). (10.4 ± 0.6 mV versus 6.5 ± 0.5 mV; $P < 0.0001$, student *t*-test (two-tailed))

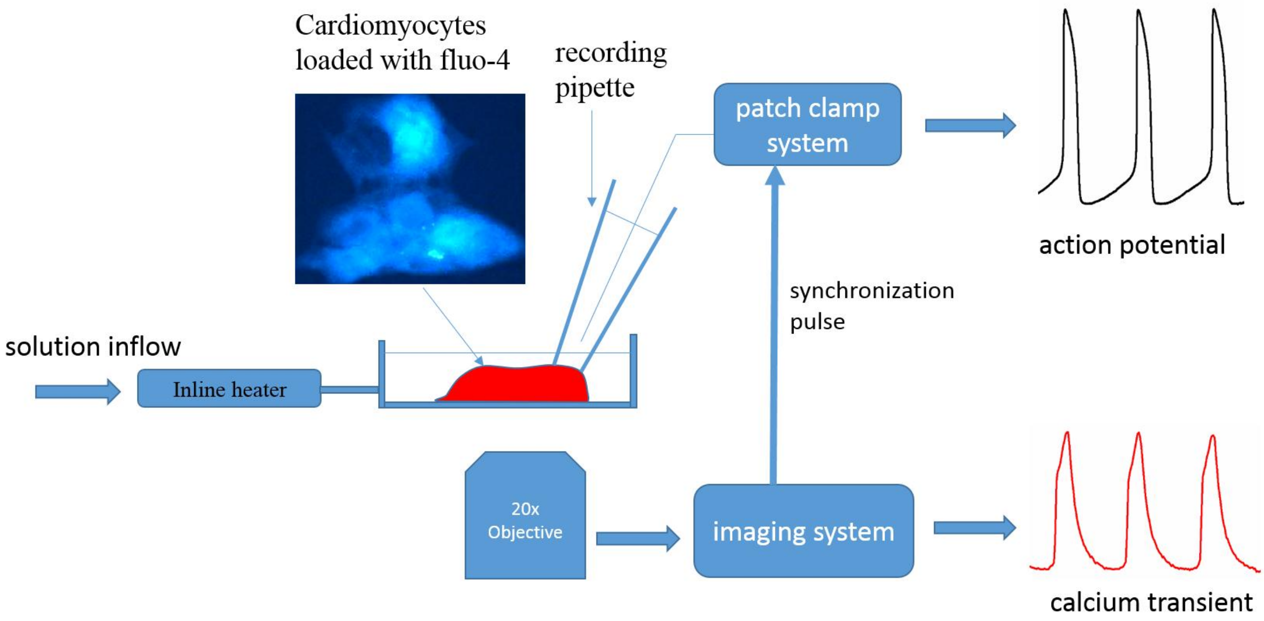

Figure S3: Patch clamp system synchronized with imaging system. Fluo-4 loaded hiPSC-CMs were continuously bath with extracellular solution. Action potentials are continuously recorded by patch clamp system. Once the calcium transients recording starts, imaging system send synchronization pulses to patch clamp system. Synchronization pulses are halted when the calcium recording stop.

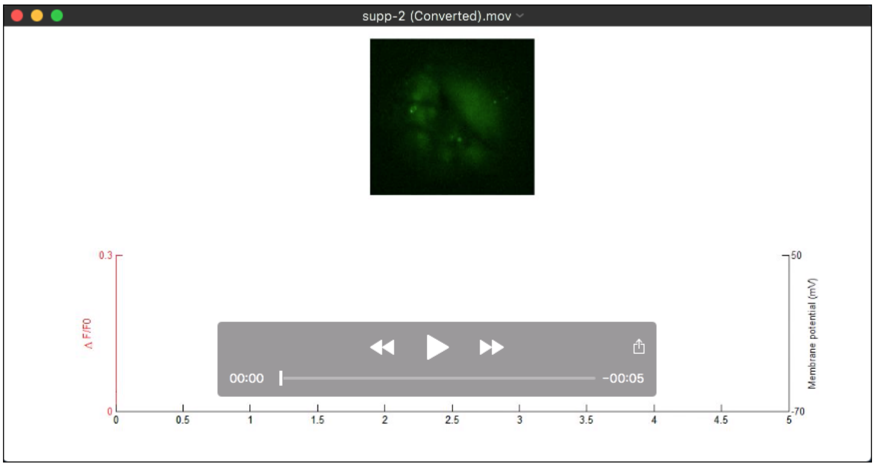

Movie 1 commentary

Live imaging of spontaneously beating hiPSC-CMs. hiPSC-CMs were loaded with Fluo-4. Patch clamp system record action potential and calcium imaging record intracellular calcium transient simultaneously.

Biology Open • Supplementary information
